# Autophagy and Rheumatoid Arthritis: Current Knowledges and Future Perspectives

**DOI:** 10.3389/fimmu.2018.01577

**Published:** 2018-07-18

**Authors:** Marta Vomero, Cristiana Barbati, Tania Colasanti, Carlo Perricone, Lucia Novelli, Fulvia Ceccarelli, Francesca Romana Spinelli, Manuela Di Franco, Fabrizio Conti, Guido Valesini, Cristiano Alessandri

**Affiliations:** Department of Internal Medicine and Medical Specialties, Sapienza University of Rome, Rome, Italy

**Keywords:** autophagy, rheumatoid arthritis, autoimmunity, apoptosis, citrullination

## Abstract

Autophagy is a degradation mechanism by which cells recycle cytoplasmic components to generate energy. By influencing lymphocyte development, survival, and proliferation, autophagy regulates the immune responses against self and non-self antigens. Deregulation of autophagic pathway has recently been implicated in the pathogenesis of several autoimmune diseases, including rheumatoid arthritis (RA). Indeed, autophagy seems to be involved in the generation of citrullinated peptides, and also in apoptosis resistance in RA. In this review, we summarize the current knowledge on the role of autophagy in RA and discuss the possibility of a clinical application of autophagy modulation in this disease.

## Introduction: Overview on Physiological Functions and Molecular Mechanism of Autophagy

**Figure 1:**
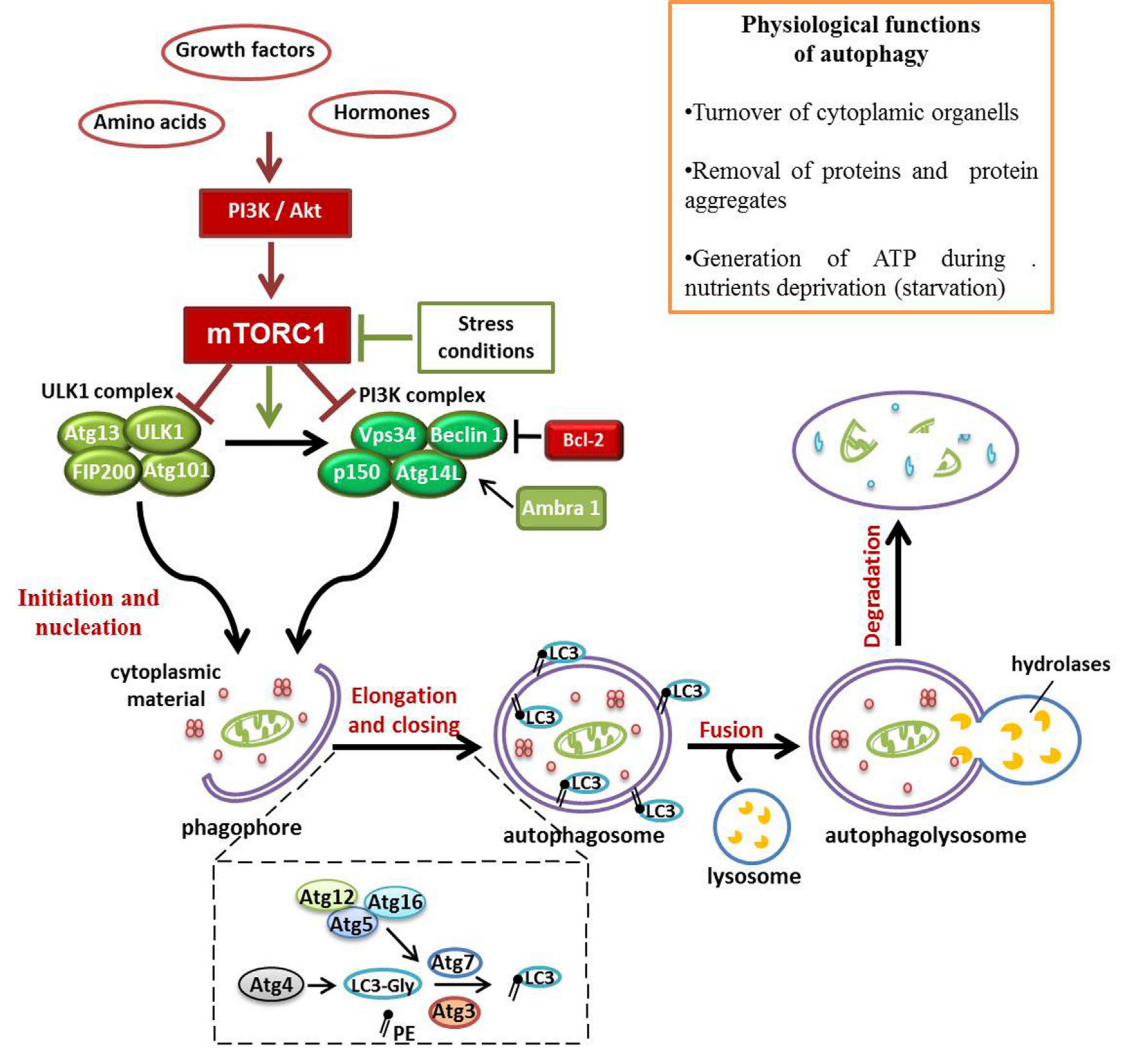
Schematic representation of signaling pathways involved in autophagy multistep regulation. Autophagy represents a fundamental physiological process, considering its role in ATP production during condition of nutrients deprivation and in controlling organelles and proteins turnover. mTOR complex 1 (mTORC1), acting as a sensor of energy levels and integrating upstream signals [phosphoinositide 3-kinase (PI3K) and Akt], is one of the most important autophagy regulators in cells. In presence of growth factors and hormones, mTORC1 inhibits autophagy activation. On the contrary, in autophagy-activated conditions, the repression of mTORC1 activity allows autophagy initiation by ULK1 and PI3K complexes recruited to the just formed phagophore. This pre-autophagosomal structure begins to engulf cytoplasmic materials and, thanks to Atg12–Atg5–Atg16L and LC3–phosphatidylethanolamine conjugated systems, it grows up and closes to generate the autophagosome. The fusion of the autophagosome with lysosome exposes the cargo to the action of lysosomal hydrolases that degrade vesicular content for recycling.

Autophagy is a degradation pathway characterized by the isolation of targeted cytoplasmic material in a typical double-membrane vesicle, known as autophagic vacuole or autophagosome (1). The subsequent fusion of the autophagosome with the lysosome ensures the correct destruction of organelles, misfolded proteins, and microorganisms, carried inside the vesicle. Despite its emerging role in human pathology, autophagy is a physiological process involved in basal organelles turnover and in the removal of proteins aggregates (2, 3). In response to the condition of cellular stress, such as growth factors and nutrients deprivation, intracellular components degraded by autophagy are recycled in order to generate ATP and sustain essential cell functions (4). Autophagy is considered a pro-survival mechanism, allowing cells to respond to injury by degrading unnecessary and dysfunctional self-components; however, this ability may become a double-edged sword (5). Three types of autophagy can be distinguished: macroautophagy, microautophagy, and chaperone-mediated autophagy. In this review, we will focus on macroautophagy (hereafter referred to as autophagy), which is the most characterized type of autophagy. Considering the crucial role of autophagy in the maintenance of cellular homeostasis, it is not surprising that several signaling-related molecules are involved in the perfect functioning of this process. Genetic screens in yeasts allowed the discovery of at least 37 autophagy-related genes (Atg) (6). Many of these genes, encoding proteins involved in autophagy and its regulation, are evolutionarily conserved in humans (7). The mammalian target of rapamycin (mTOR) complex 1 (mTORC1) regulates the activation of autophagy machinery, acting as a sensor of energy levels and integrating upstream signals deriving from other pathways, including the phosphoinositide 3-kinase (PI3K)-Akt. As displayed in Figure 1, in the presence of aminoacids and growth factors, mTORC1 represses autophagy by inhibition of Vps34 and ULK1 complexes. On the contrary, in low nutrients state, defined as starvation, the dissociation of mTORC1 from the induction complex triggers autophagy (8, 9). The autophagosome derives from a double-membrane pre-autophagosome structure called phagophore, which seems to originate from different sources, including plasma membrane (10), endoplasmic reticulum (ER) (11), and Golgi complex, in mammalian cells (12). Phagophore nucleation requires the activity of class III phosphatidylinositol 3-kinase (PI3K-III) complex containing Beclin-1 (a mammalian homolog of yeast Atg6), hVps34, p150 (a mammalian homolog of yeast Vps15), and Atg14-like protein (Atg14L) (13). The autophagy promoting function of Beclin-1 is influenced by the antiapoptotic protein Bcl-2; in fact, when Beclin-1 is bound to Bcl-2, autophagy is inhibited; instead, the dissociation from Bcl-2 allows Beclin-1 to interact with PI3K-III complex, and to activate autophagy (14). On the contrary, Beclin-1 regulated autophagy protein 1 (AMBRA1) is a positive regulator of Beclin-1-dependent autophagy; thanks to its capacity to create a link between cytoskeletal motor proteins and class III PI3K complex (15, 16). Two ubiquitin-like conjugation systems, Atg12–Atg5–Atg16L and microtubule-associated protein 1 light-chain 3 (LC3)–phosphatidylethanolamine (PE), mediate the second step of autophagy, which concerns the expansion and closure of the autophagosome (17). In the first system, the enzymes E1-like Atg7 and E2-like Atg19 promote the covalent association of Atg12 to Atg5 (Figure 1). Subsequently, Atg16 binds to the complex to form the heterotrimer Atg16-Atg12-Atg5, this organization at the level of the outer portion of autophagosomal membrane, mediates the curvature of the growing membrane and also participates in the association of LC3 to PE (18). LC3 is cleaved by the cysteine protease Atg4 to produce the cytosolic form LC3-I, which, after being activated by Atg7, is transferred to Atg3 in order to be changed in the conjugate form with PE, named LC3-II (Figure 1). LC3-II is thus the most commonly used marker to test autophagic activity, being then the only protein that remains stably associated with the autophagosome in maturation (6). Upon being formed, the autophagosome fuses with the lysosome to generate the autophagolysosome, in which the vesicular content is degraded by lysosomal hydrolases. Finally, products of degradation, such as aminoacids and lipids, are exported from autophagy-related compartments to the cytoplasm to be recycled and generate new macromolecules (19).

## Emerging Roles of Autophagy in Human Pathology

Since the first observation of autophagy, more than 50 years ago, there has been a growing interest in studying this mechanism, and deregulated autophagy has been recently connected with the pathogenesis of several diseases. Aging is not properly considered a disease; however, it is associated with different pathological conditions. In the last period of human life, cells undergo several changes, including DNA mutations, damages at several other molecules, and accumulation of protein aggregates. Several studies have demonstrated that autophagy activation protects from aging. In fact, not only autophagy levels decrease with age but also overexpression of Atg proteins contributes to improve life span in a human model of aging *in vitro* and in mouse models *in vivo* (20–22). As already mentioned, one of the most important functions of autophagy is the degradation of misfolded proteins, so in neurons, the failure of autophagy can contribute to neurodegeneration (23). It can occur in Parkinson’s Disease, a neurodegenerative disorder characterized by α-synuclein accumulation in the brain. In a study published by our group, we demonstrated that autophagy inhibition by 3-methyladenine (3-MA) and by Atg5 knocking down in lymphocytes lead to a significant increase of α-synuclein levels (24).

Moreover, autophagy seems to be linked also to cancer; however, this relationship is still controversial. The removal of mitochondria, source of reactive oxygen species (ROS), performed by autophagy, certainly protects cells from DNA mutations and prevents cellular transformation. It has also been demonstrated that deletion of the autophagic gene Beclin-1 may cause development of various malignancies in mouse models (25). Autophagy is also involved in the degradation of intracellular pathogens, which represent a source of proteins involved in tumorigenesis (26). Despite its role in the maintenance of genomic stability, many studies indicate a tumor-supporting function of autophagy, allowing tumor cells to respond to stress stimuli, such as nutrients deficiency and hypoxia, thus extending their lifespan. Furthermore, it has been demonstrated that autophagy-deficient tumors are more sensitive to several chemotherapeutic agents (27, 28). In this case, autophagy promotes the survival of cancer cells and protects them from the action of drugs that induce apoptosis. Although research in this field is just at the beginning, an encouraging number of works suggest that defects in the autophagy mechanism may be involved in the pathogenesis of autoimmune diseases (29). In our previous works, we focused on systemic lupus erythematosus (SLE), showing that factors present in the serum of SLE patients, probably antibodies, are able to induce autophagy in T lymphocytes from healthy donors, but not in T lymphocytes from patients with SLE. We speculated that chronic exposure to specific autoantibodies, as occurs in SLE, could lead to the selection of autophagy-resistant T lymphocytes (30). Some of these autoantibodies could be directed to D4GDI, a regulator of Rho proteins activation. More recently, we also identified IgGs directed to D4GDI in sera from patients with SLE (31). On the contrary, very little is known on the role of autophagy in the pathogenesis of rheumatoid arthritis (RA) and other autoimmune rheumatic diseases, thus making research on autophagy in autoimmune conditions a very intriguing field.

## Autophagy in Autoimmunity Affair: Role of Autophagy in RA

Rheumatoid arthritis is a chronic autoimmune disease affecting not only the joints but also other organs including heart, vascular system, lungs, and skin. Environmental and genetic factors both lead to immune cells activation against self-antigens and production of autoantibodies, such as anticyclic citrullinated peptide (anti-CCP) antibodies (Abs), pathognomonic markers of this disease (32, 33). Several immune cells are involved: T and B cells, macrophages, synovial fibroblasts, chondrocytes, and osteoclasts, which lead to the release of different inflammatory mediators, sustaining the chronic inflammatory response of the disease (34).

### Autophagy in Immunological Tolerance

Many studies demonstrated autophagy’s contribution to the presentation of cytosolic antigens in association with MHC class II molecules, playing an important role not only in the acquired immune response but also in the maintenance of self-tolerance (35). Mechanisms of central (in the primary lymphoid organs) and peripheral tolerance (in peripheral tissues) physiologically prevent immune responses to self-antigens (36). During T cells development in the thymus, the recognition of peptide–MHC molecules on the surface of thymic epithelial cells (TECs) ensures that only thymocytes restricted to MHC molecules, and specific for non-self (foreign) antigens, will survive and continue their maturation.

Emerging evidence indicates that autophagy contributes to the maintenance of the central tolerance mechanism (37). Mizushima and colleagues (38) found high autophagy levels in TECs, suggesting a possible involvement of autophagy in the formation of the lymphocytes repertoire during thymic selection. According to this hypothesis, it was recently revealed that there had been an alteration in the selection of the T cell receptor (TCR) restricted to MHC class II in mice transplanted with Atg5^−/−^ thymus. Autophagy defects, in association with a consequent loss of self-tolerance, could be the reason of multiple signs of autoimmunity reported in these animals (39). However, Sukseree and colleagues demonstrated that autophagy suppression did not affect the selection of lymphocytes repertoire in TECs (40). These two opposite results probably depend on the different approach used to inhibit autophagy in the thymus, thus further investigations are necessary.

The involvement of autophagy in the presentation of self-antigens to immature T cells in the thymus was first analyzed by Kasai and colleagues, who showed a colocalization of LC3-II with the lysosomal compartment in which MHC–peptide complexes are formed (35). More recently, Aichinger et al. demonstrated that autophagy is essential for endogenous antigen-loading onto MHC class II of TECs for negative selection (41).

### Autophagy in Joint Destruction

Although for many years the role of Th1 cells has been considered predominant in RA, recently, a crucial role of Th17 cells is also emerging (42). This cell subset is a primary source of the pro-inflammatory cytokine IL-17, which acts in synergy with TNF-α and IL-1, contributing to the bone destruction. In this context, receptor activator of nuclear factor kB (NF-kB) ligand (RANKL) produced by activated T and B cells and fibroblasts, by binding to its receptor RANK, expressed in monocyte–macrophage lineage, stimulates the differentiation of osteoclast precursor cells in mature osteoclasts (43). Most recent findings suggest a possible involvement of autophagy in osteoclastogenesis. Specifically, hypoxia, which is an autophagy-activating stimulus, seems to be able to stimulate maturation of osteoclasts (44); moreover, it has been demonstrated that the inhibition of autophagy blocked osteoclastogenesis in mouse monocyte/macrophage cell lines (45). In another work, it has been shown that treatment with RANKL caused up-regulation of autophagy markers and the knockdown of autophagy substrate p62 decreased the expression of genes involved in the osteoclastogenesis process (46). In experimental arthritis mouse models, the inhibition of autophagy reduced signs of bone erosion and the number of osteoclasts, suggesting a key role of autophagy in bone tissue degradation (47). In this regard, drugs that downregulate autophagy may be used to prevent bone resorption in RA patients.

### Autophagy as a Protective Mechanism Against Apoptosis

Autophagy acts promoting cell survival under conditions of nutrients deficiency, while apoptosis is a fundamental programmed cell death mechanism, thus the relationship between these two processes influences cell fate. Moreover, through the elimination of damaged mitochondria, autophagy also participates in the reduction of ROS and damaged DNA, thus preventing the development of apoptosis (48). In several studies in which autophagy was suppressed by knocking down autophagy genes, cell death was not inhibited, but increased, indicating the prominent role of autophagy as a cell survival mechanism (49).

Different molecules are common to both cellular mechanisms. As already discussed, family members of Bcl-2, well known as apoptosis regulators, are able to modulate also autophagy by inhibition of Beclin-1 (14). It has been demonstrated that caspase-dependent cleavage of Beclin-1 and its subsequent localization to mitochondria promotes the release of proapoptotic factors from these organelles (50). The balance between cell survival and cell death is essential in regulating immune cells destiny and it seems to have a crucial role in RA pathogenesis and progression. One of the most important apoptosis functions consists in the extinguishing of inflammation, by blocking an excessive immune cells activation and cytokines production. In this regard, a reduction of apoptosis rate and apoptotic mediators was found at the synovial level, indicating a downregulation of apoptosis in RA (51, 52). In fact, RA synovial fibroblasts are subjected to a complex pattern of molecular changes, including alterations in the expression of signaling pathways that lead to an aggressive and invasive phenotype (53). The progressive bone and cartilage destruction is attributable to resistance of synovial fibroblasts to apoptosis induction, and several intracellular processes, including autophagy, could take part in this phenomenon (54). As already discussed, there is a controversial crosstalk between autophagy and apoptosis; autophagy induction could be a potential mechanism by which RA cells protect themselves from apoptosis, increasing thus their lifespan. In support of this hypothesis, ER stress caused higher autophagy activation in synovial fibroblasts obtained from patients with RA than in those from osteoarthritis (OA) patients, and RA-fibroblast-like synoviocytes (FLS) appeared to be more resistant to cell death induction (55). Moreover, an inverse correlation between autophagy and apoptosis in synovial tissues from RA patients was found, indicating an involvement of autophagy in the apoptosis-resistant phenotype of RA synoviocytes (56, 57). Recently, immune-histochemical and molecular analysis of autophagy-related molecules on synovial biopsies showed increased levels of Beclin1, Atg5, and LC3-II in RA compared to OA patients (58). It is important to note that TNF-α is not only a potent modulator of inflammatory response in RA but also an apoptosis-activator molecule, inducing autophagy in different cell types including skeletal muscle, atherosclerotic vascular smooth cells, and also RA synoviocytes (59, 60). Connor and co-authors studied the effect of TNF-α on protein degradation, demonstrating that in RA synovial fibroblasts, TNF-α stimulates the conversion of LC3-I to LC3-II but not the activation of proteasome complex (61). A research work by Xu and colleagues revealed a connection between autophagy hyperactivation and methotrexate (MTX) resistance, by showing that RA-FLS undergo higher levels of MTX-induced apoptosis when autophagy is inhibited (62). More recently, in a collagen-induced arthritis (CIA) rat model, it has been demonstrated that inhibition of autophagy alleviated synovial inflammation and promoted synovial cell apoptosis through the regulation of PI3K/AKT pathway (63). These data confer to autophagy an important protective role against apoptosis; for these reasons, therapy based on autophagy repression might have a beneficial effect in RA.

### Autophagy in Lymphocytes Homeostasis

Peripheral immune cells play an important role in the perpetuation of autoimmunity by sustaining systemic inflammation status and by participating in the extension of joint destruction mechanisms. Many studies demonstrated that autophagy allows T and B lymphocytes to survive in conditions of nutrients deprivation or during stress stimuli (64). Mice lacking Atg5 do not survive and have a reduction of peripheral T cells, showing how autophagy is essential for their survival (65). Since cytoplasmic calcium levels are essential for TCR-signaling pathways activation, autophagy-dependent calcium flux regulation could influence T lymphocytes activation. It has been demonstrated that CD4^+^ and CD8^+^ Atg5^−/−^ cells are not able to properly proliferate following TCR stimulation (65). Moreover, the inhibition of autophagy causes defects in T cell activation. In fact, deletion of Atg7 results in decreased in IL-2 mRNA level and ATP generation, suggesting that autophagy is required to ensure appropriate energy level for T cell activation (66). Similar data were obtained also on B lymphocytes, demonstrating that autophagy is essential for the maturation process and for the subsequent maintenance of B lymphocytes repertoire in the periphery (67, 68).

Systemic autoimmune diseases such as RA are characterized by secretion of pathogenic autoantibodies by plasma cells (PCs), and an increase of this phenomenon seems to be associated with autophagy defects (69). First of all, autophagy seems to be involved in “PC differentiation program” since it has been found to be activated during this process (70). The absence of the autophagic gene Atg5 does not alter B lymphocytes differentiation, but these cells secrete a larger amount of Abs compared to the wild-type counterpart (71). On the other hand, the suppression of autophagy makes PCs more susceptible to cell death, stopping in this way the persistent Abs secretion. Conway and colleagues obtained similar results in the same mouse model, underlying a crucial role of autophagy in PCs homeostasis (72).

Studies on the role of autophagy in lymphocytes isolated from RA patients are scarce and yet contradictory. Yang and colleagues explored the metabolic activity of RA T cells, showing a defect of autophagy in these cells related to a deficiency of PFKFB3, a regulatory glycolytic enzyme (73). Opposite data were recently published by van Loosdregt and co-authors. They demonstrated that CD4^+^ T cells from RA patients treated with hydroxychloroquine (HCQ) showed increased levels of LC3-II and autophagosomes number compared with cells isolated from healthy donors (74). Autophagy hyperactivation was found in CIA mouse model both in CD4^+^ T cells and at inflammatory sites. Moreover, a reduction of arthritis signs was noticed after the animals were injected with the autophagy inhibitor HCQ (74).

In conclusion, autophagy could maintain autoreactive T and B cells populations sustaining RA chronic inflammatory response, but more experimental evidences are needed to confirm this hypothesis.

### Autophagy and Citrullination

Citrullination, chemical conversion of arginine in citrulline by the action of peptidylarginine deiminase (PAD) enzymes (75), has a crucial role in RA pathogenesis and the presence of autoantibodies directed against citrullinated peptides is often associated with a poor prognosis. Anti-CCP Abs target certain epitopes of citrullinated autoantigens and have a crucial role in RA development due to their pathogenetic potential (76). In fact, anti-CCP Abs purified from RA patient is capable of causing not only *in vitro* differentiation of human osteoclasts but also bone loss, when they are injected into mice (77). The contribution of autophagy to the presentation of citrullinated peptides and to the generation of anti-CCP Abs seems to be relevant in RA. Ireland and colleagues showed that antigen-presenting cells (APCs) need autophagy to successfully perform citrullinated proteins presentation, but not the unmodified antigens presentation, and this process is stopped following autophagy inhibition (78). Moreover, since PAD enzyme was found to be expressed in autophagy compartment, and classical pro-autophagic stimuli, such as nutrients deprivation, promoted the presentation of citrullinated peptides in B cells, the authors thought that citrullination could represent a “biochemical marker of autophagy” (79). In a more recent study, in FLS from RA patients, the levels of some citrullinated protein, such as vimentin and α-enolase, increased after treatment with the autophagy inducer rapamycin (80). Furthermore, for the first time, a direct correlation between LC3-II levels and anti-CCP titers was found in monocytes from early active RA patients. These experimental evidences highlight that autophagy activation may participate to the break of self-tolerance by sustaining generation of citrullinated peptides.

### Oxidative Stress

Production of ROS and reactive nitrogen species is triggered by different elements such as metabolism and endogenous inflammation, and exogenous factors including UV light and ionizing radiation. Despite the physiological production of these molecules, their accumulation can be deleterious for cell homeostasis, leading to DNA mutation and could stimulate different molecular pathways, including NF-kB activation, with consequent cytokines production and inflammation (81). Furthermore, protein structural changes induced by ROS are able to modify “primitive” antigen and to form new peptides that can trigger an autoimmune response (82). For these reasons, an imbalance in oxidative stress regulation plays a crucial role in inflammatory autoimmune disorders also by modulating cell fate mechanisms. Mitochondria are the most important source of ROS and autophagy-mediated mitochondria degradation, called mitophagy, ensures correct balance of oxidative species levels in cells. However, the relationship between autophagy and oxidative stress seems to be very complex in the context of RA, where not only total oxidative status is higher in patients than in healthy control but also neutrophils ROS levels correlate positively with disease activity (83, 84). Since it has been demonstrated an involvement of ROS in autophagosome formation by regulation of Atg4 function (85), ROS-mediated autophagy induction could contribute to the resistance of apoptosis found in synovial and peripheral RA T cells and to the generation of citrullinated peptides in APCs, both these aspects will be important to deepen in future studies.

**Figure 2:**
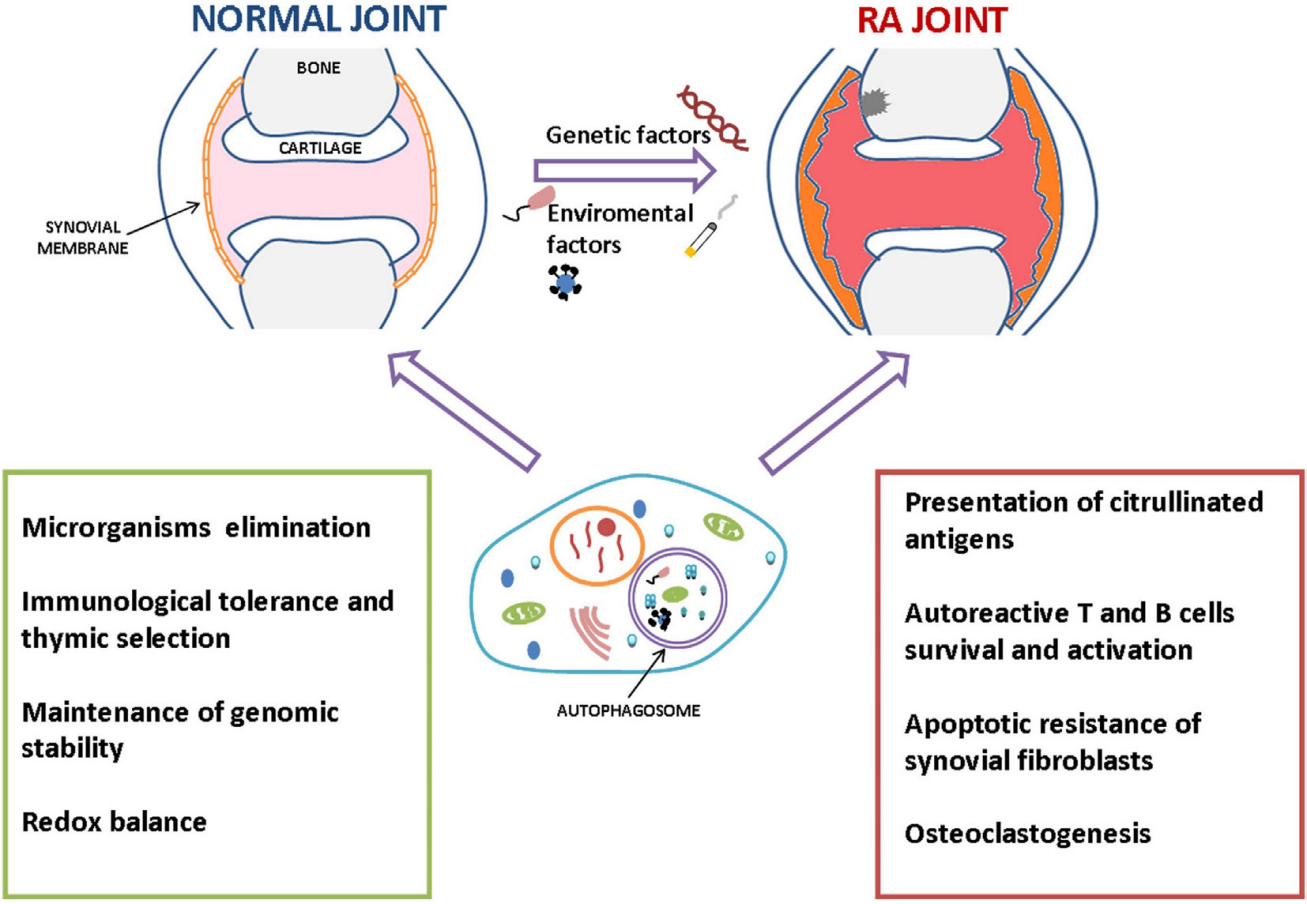
Summary of the possible roles of autophagy in the pathogenesis and progression of rheumatoid arthritis (RA). Autophagy could prevent the development of autoimmunity scenario by eliminating intracellular pathogens and maintaining immune cells homeostasis, including regulation of immunological tolerance mechanisms. However, once it occurs, autophagy could take part in RA progression in several ways, e.g., participating in perpetuation of autoimmune response promoting the survival of inflammatory and autoreactive cells, cytokines production, and presentation of citrullinated antigens.

The possible roles of autophagy in RA pathogenesis and progression are summarized in Figure 2.

## Targeting Autophagy in RA Therapy

Considering the key role of autophagy not only in innate and adaptive immune regulation but also in immune system cells homeostasis, it is not surprising that autophagy modulation might be an attractive therapeutic choice in RA. As described in our previous review, rapamycin, an autophagy activator with mTOR inhibitory effects, led to a decrease of disease activity in a small group of SLE patients refractory to traditional treatments (29); at present, a clinical trial is testing the possible use of autophagy modulator in SLE treatment (https://clinicaltrials.gov/ct2/show/study/NCT00779194). Both in SLE and RA, drugs that are able to modulate autophagy, such as CQ and HCQ, are currently in use in the clinical practice showing a high effectiveness (86). It has been demonstrated that CQ is able to inhibit antigen presentation to T cells (87) and the differentiation of osteoclast precursors into mature osteoclasts *in vitro* and *in vivo* (88); in both these processes, autophagy is actively involved.

As previously discussed, although TNF-α was found to induce autophagy in different cell types directly associated with RA pathogenesis, till now, the effect of anti-TNF drugs on autophagy has not been investigated. TNF-mediated autophagy may have a role in the resistance to apoptosis, well documented in the disease, and the blocking of autophagy induction by anti-TNF drugs may reactivate apoptosis. This hypothesis is validated also by a study of Catrina and colleagues, who demonstrated an activation of synovial apoptosis after 8 weeks of treatment with either etanercept or infliximab (89, 90).

A future therapeutic approach based on autophagy suppression in RA might include 3-MA, a chemical compound that inhibits autophagy at an early stage of autophagosome formation, blocking the signaling of PI3K. This pathway has been used in experiments to analyze how autophagy inhibition can lead to beneficial effect in RA, including reduction of citrullinated peptides presentation and the reactivation of apoptosis pathways (57). Data on the systemic effect of 3-MA are limited, but a recent study showed an atheroprotective role of 3-MA, probably related to a downregulation of inflammation, in ApoE-deficient mice (91).

The importance of the balance between autophagy and apoptosis in the resistance to treatment in RA patients has been recently proposed by Xu and colleagues (62). The authors showed that MTX, commonly used in the treatment of RA, is able to induce autophagy in synovial cells protecting them from apoptosis. In fact, the inhibition of autophagy by Beclin-1 siRNA caused an increased death of these cells by apoptosis. Taking all of these considerations, a therapy based on combination of MTX and an autophagy inhibitor in RA has been proposed.

Glucocorticoids have been widely used in the treatment of autoimmune disorders for its anti-inflammatory and immunosuppressive action, although one of the dark effects of this therapy consists in a substantial risk of bone injury. A recent study demonstrated a pro-autophagic effect of glucocorticoid on bone marrow mesenchymal stem cells (BMSCs), concluding that autophagy activation sustained the proliferative potential of BMSCs by protecting them from apoptosis (92). Shen et al. showed that the induction of autophagy by rapamycin blocked the dexamethasone-induced apoptosis in meniscal cells, while the treatment with the autophagy inhibitor 3-MA increased the number of apoptotic cells (93). According to these results, other studies on condrocytes suggest a beneficial effect on bone loss by induction of autophagy contrasting glucocorticoid-induced apoptosis (94).

Abnormalities in PI3K/AKT/mTOR axis have been found in active RA patients and the activation of this pathway has been associated with an excessive activation, proliferation, and survival of T and B cells and apoptosis resistance in RA synoviocytes (95). The suppression of mTOR signaling may be another way to treat RA by modulating autophagy. In a multi-center study involving 121 RA patients, it was found that there was a greater response to the therapy in the group of patients treated with the mTOR inhibitor everolimus plus MTX than the MTX alone, suggesting that autophagy modulators may be added to standard therapy to increase the effectiveness of the therapy (96). This result was corroborated by Cejka and colleagues, who found a reduction in osteoclast number and bone erosions in TNF-transgenic mice treated with sirolimus or everolimus (97). Recently, a clinical trial compared the therapeutic response of temsirolimus (CCI-779) at three different concentrations with placebo in active RA patients (https://clinicaltrials.gov/ct2/show/record/NCT00076206). Considering the pleiotropic role of mTOR signaling in cell metabolism, it is important to note that the effects of mTOR inhibition might not be due as much to autophagy inhibition but rather to the shutdown of other related pathways. In fact, and not surprisingly, personalized pharmacological mTOR blockade has been proposed for the treatment of several non-immune and immune-related disorders (98). To conclude, there are several autophagy modulators in use or under investigation in the management of RA therapy (Table 1). This list includes both inducers and inhibitors of autophagy, reflecting the controversial role of this process in the pathogenesis of RA. Considering the intricate signaling pathways regulating autophagy, the pleiotropic activity of some of these drugs still represents the most enigmatic aspect. In fact, by acting on different substrates, they can produce opposite signals associated to autophagy activation. Moreover, *in vitro* studies revealed how experimental conditions, timing, and cell types can influence autophagy-associated results.

**Table 1:** Autophagy modulators of clinical relevance in rheumatoid arthritis (RA).

Regulators of autophagy	Use/not in use in RA treatment	Effects on RA mediated by autophagy modulation	Reference
**Inducers**			
Rapamacin	Not in use	Promotion of cell survival; citrullination	(96–98)
Glucocorticoids	In use	Chondrocytes apoptosis protection	(93, 94)
Methotrexate	In use	Resistance to RA-FLS apoptosis	(62)

**Inhibitors**			
Chloroquine	In use	Inhibition of antigen presentation	(87, 88)
3-MA	Not in use	Reduction of citrullinated peptides presentation; apoptosis induction	(57, 91)
Anti-TNF drugs (?)	In use	Apoptosis reactivation	(89, 90)

## Conclusion and Perspectives

The introduction of biologic agents has revolutionized the clinical approach to RA; however, research of new therapeutic targets appears to be essential to improve the response to therapy. Autophagy is a crucial physiological process and its functions are strictly related to tissue and environmental conditions. Increasing evidences point to autophagy as a driving mechanism of autoimmune diseases. In RA, autophagy activation was found to be essential for the survival of inflammatory cells such as synoviocytes and lymphocytes and has an important role in citrullination and osteoclastogenesis. However, repression of autophagy could expose patients to premature aging, infections, and development of malignancy. Currently, compounds that modulate autophagy pathway are approved for the management of the disease, but long-term effects must be evaluated in order to analyze whether autophagy modulation can interfere with other biological phenomena.

## Author Contributions

MV and CA designed and wrote the review. CB and TC finalized the table and figures and drafted the manuscript. CP, LN, FUC, FS, MF, FAC, and GV drafted the manuscript and provided useful suggestions. All authors read and approved the final manuscript.

## Conflict of Interest Statement

The authors declare that the research was conducted in the absence of any commercial or financial relationships that could be construed as a potential conflict of interest.

## Abbreviations

Abs, antibodies; AMBRA1, Beclin-1 regulated autophagy protein 1; anti-CCP, anti-cyclic citrullinated peptide; APC, antigen-presenting cells; Atg, autophagy-related genes; BMSCs, bone marrow mesenchymal stem cells; CIA, collagen-induced arthritis; ER, endoplasmic reticulum; FLS, fibroblast-like synoviocytes; HCQ, hydroxychloroquine; LC3, microtubule-associated protein 1 light-chain 3; mTOR, mammalian target of rapamycin; mTORC1, mammalian target of rapamycin complex 1; MTX, methotrexate; OA, osteoarthritis; PAD, peptidylarginine deiminase; PCs, plasma cells; PE, phosphatidylethanolamine; PI3K-III, class III phosphatidylinositol 3-kinase; RA, rheumatoid arthritis; RANKL, receptor activator of nuclear factor kB (NF-kB) ligand; ROS, reactive oxygen species; SLE, systemic lupus erythematosus; TECs, thymic epithelial cells.
